# Level of systolic blood pressure within the normal range and risk of cardiovascular events in the absence of risk factors in Chinese

**DOI:** 10.1038/s41371-021-00598-1

**Published:** 2021-09-03

**Authors:** Chunpeng Ji, Na Wang, Jihong Shi, Zhe Huang, Shuohua Chen, Guodong Wang, Shouling Wu, Jost B. Jonas

**Affiliations:** 1 Department of Cardiology, Kailuan General Hospital, North China University of Science and Technology, Tangshan, China; 2 Department of Cardiology, The Second Hospital of Qinhuangdao, Qinhuangdao, China; 3 Health Care Center, Kailuan Medical group, Tangshan, China; 4 Department of Ophthalmology, Medical Faculty Mannheim of the Heidelberg University, Mannheim, Germany; 5 Institute of Molecular and Clinical Ophthalmology Basel, Basel, Switzerland; 6 Privatpraxis Prof Jonas und Dr Panda-Jonas, Heidelberg, Germany

**Keywords:** Hypertension, Diagnosis

## Abstract

The risk of cardiovascular disease (CVD) at currently defined normal systolic blood pressure (SBP) levels in individuals without CVD risk factors is not well examined. We evaluated whether higher systolic blood pressure within the range considered normal is associated with a higher CVD risk in Chinese without traditional CVD risk factors. The community-based study included 25,529 individuals (mean age:47.3 ± 12.3 years;range:18–95 years) with a baseline SBP of 90–129 mmHg, who were free of CVD and traditional CVD risk factors, and who were re-examined in biennial intervals. During a mean follow-up of 10.6 ± 1.49 years (maximum. 11.5 years), 847 CVD events occurred. CVD incidence per 1000 person-years increased with higher baseline SBP levels (SBP,90–99 mmHg:1.45;100–109 mmHg:2.15;110–119 mmHg:3.06; and 120–129 mmHg:3.80). After adjusting for CVD risk factors, the categorical Cox regression suggested that the CVD risk was not statistically significant for study participants with a baseline SBP level of 100–109 mmHg, 110–119 mmHg, and 120–129 mmHg compared with those with a baseline SBP level of 90–99 mmHg. If CVD risk factors including blood pressure categories which developed during follow-up were included in a time-dependent Cox regression analysis, the normal baseline SBP was still not associated with incident CVDs. A SBP between 90 and 129 mmHg was not associated with an increased CVD risk in a healthy population.

## Introduction

As an important risk factor for cardiovascular diseases (CVDs), high blood pressure (BP) has been a leading cause of disability and death [1, 2]. In the Global Burden of Diseases Study 2019, high systolic BP (SBP), defined by a theoretical minimum-risk exposure level of 110–115 mmHg, accounted for 10.8 million deaths (19.2% of all deaths) [1, 3]. With an improvement in BP control, more than 60% of incident CVD events occurred in the normotensive population (SBP/diastolic BP [DBP] <140/90 mmHg) [4]. This also led to a shift in the focus of BP-related research.

A recent meta-analysis including 61 cohort studies showed that the CVD risk increased starting from a relatively low BP level (SBP/DBP: 115/75 mmHg) [5]. The SBP intervention trial (SPRINT) found that intensive antihypertensive treatment (lowering SBP/DBP to <120/80 mmHg), as compared with standard antihypertensive treatment with a lowering of the SBP/DBP to <140/90 mmHg, reduced the risk of CVD and all-cause death [6]. Based on the results of these and other studies, the 2017 American Heart Association (AHA)/American College of Cardiology (ACC) guidelines reduced the SBP level defining hypertension from 140 to 130 mmHg [7]. It has remained unclear whether the association between the SBP level and the CVD risk is valid also among healthy normotensive adults without traditional cardiovascular risk factors. The multi-ethnic study of atherosclerosis (MESA) recently reported that in 1457 normotensive participants without conventional cardiovascular risk factors, the CVD risk increased during an average follow-up of 14.5 years by 53% for every 10 mmHg increase in the baseline SBP, ranging between 90 and 129 mmHg [8]. It suggested a dose-effect rather than a J-shaped or U-shaped relationship between the SBP level and CVD risk in a healthy population. Since such results are of clinical and general importance and since the CVD incidence and its risk factors differ between regions and societies, we performed this study to re-investigate the association between the baseline SBP level within the normal range and the CVD risk in a normotensive population without conventional cardiovascular risk factors in China.

## Methods

The Kailuan study (registration number: ChiCTR-TNC-1100148) is a prospective community-based cohort study that was performed in the community of Kailuan in the industrial city of Tangshan in the Chinese province of Hebei [9–11]. The Ethics Committees of Kailuan General Hospital confirmed that the study followed the guidelines of the Helsinki Declaration and approved it. All participants signed a written informed consent. The study participants were employees and retirees of the Kailuan Group Company. The latter is a coal mining industry in Tangshan. At baseline of the study between June 2006 and October 2007, we examined the study population of 101,510 individuals (81,110 men) with an age ranging between 18 and 98 years. All participants underwent re-examinations in the clinical examination rooms in 2-year intervals. The examinations included an interview with standardized questions on demographic, socioeconomic and clinical parameters, determination of anthropometric parameters, measurement of BP, biochemical analysis of blood samples, and other measures. Mercury sphygmomanometers were used to measure the BP of the left arm with a cuff of appropriate size following the recommended standard procedures. After the participants had rested in a chair for at least 5 min, we took the measurements at 5 min intervals. We used the average of three readings for further data analysis. The eGFR was calculated using the Chronic Kidney Disease Epidemiology Collaboration (CKD-EPI) formula [12].

For the present investigations, we included all participants of the Kailuan Study with a normal SBP (90–129 mmHg) and without traditional CVD risk factors at the baseline visit in 2006–2007. We excluded participants with missing values of SBP, serum concentrations of low-density lipoprotein cholesterol (LDL-C), high-density lipoprotein cholesterol (HDL-C) and glucose (*n* = 1557), individuals with a SBP < 90 mmHg (*n* = 318) or a SBP ≥ 130 mmHg (*n* = 51,156), persons taking medications against hypertension (*n* = 1762), individuals with dyslipidemia (defined by a LDL-C level ≥ 160 mg/dL, HDL-C level < 40 mg/dL, or reported use of cholesterol-lowering medications, *n* = 4455), patients with diabetes (glucose level ≥126 mg/dL or use of blood glucose–lowering medications, *n* = 2269), current smokers (*n* = 14,152), and individuals with a history of CVD (*n* = 312). It left a total of 25,529 participants, who were followed up till December 31st, 2017 or up to the time at which a CVD event including myocardial infarction and stroke, or death occurred, whichever came first. We defined CVD risk factors based on conventional categorical CVD risk factors as published previously [13–15].

The main outcome parameter was the incident CVD event including myocardial infarction, heart failure, cerebral infarction, and cerebral hemorrhage. We defined an CVD event as described previously [10]. All study participants were linked to the Municipal Social Insurance Institution and the Hospital Discharge Register which allowed the detection of an incident CVD. To identify potential additional study participants with CVD events, we reviewed the discharge lists from the 11 Kailuan hospitals during the study period from 2006 to 2017, and we asked the study participants at each re-examination about a previous CVD event. For all suspected CVD events, three experienced masked physicians reviewed the medical records and adjudicated. Incident myocardial infarction was diagnosed according to the criteria of the World Health Organization on the basis of clinical symptoms, changes in the serum concentrations of cardiac enzymes and/or biomarkers, and electrocardiogram results [16, 17]. The diagnosis of heart failure was based on clinical symptoms and objective evidence (such as abnormalities of echocardiography, chest radiographs, B-type natriuretic peptide, etc.) [18]. Stroke was diagnosed according to the World Health Organization criteria [19].

### Statistical analysis

We divided the study population into four groups based on their SBP levels, namely 90–99 mmHg, 100–109 mmHg, 110–119 mmHg, and 120–129 mmHg. The incidence density of CVD events was calculated as number of cases divided by person-years. To examine the association between the baseline SBP (continuous) and incident CVD, we performed a restricted cubic spline interpolation with a reference value of a SBP of 110 mmHg allowing for three knots, which were selected based on Harrell’s recommended percentiles at SBP values of 98.7 mmHg (5th percentile), 116.7 mmHg (50th percentile), and 126 mmHg (95th percentile) [20]. This restricted cubic spline interpolation was adjusted for age, sex, alcohol consumption status (never and past, or current (i.e., ≥once/day)), ever smoking (yes/no), education level (elementary school, high school, or above), physical exercise (none, occasionally, or frequently (i.e., ≥once/week)), prediabetes (yes/no), family history of CVD (yes/no), DBP, body mass index, estimated glomerular filtration rate (eGFR), and serum concentrations of total cholesterol, high-density lipoproteins-C, glucose serum concentration, uric acid, and C-reactive protein.

The Cox regression was used to estimate hazard ratios (HRs) and 95% confidence intervals (CIs) for CVD in the different SBP groups with an SBP level of 90–99 mmHg as the reference. Model 1 was unadjusted, model 2 was adjusted for age and sex, model 3 was further adjusted for the other covariates mentioned above, and model 4 was a competing risk model of death which adjusted for all the confounders in model 3. We further examined the interaction between the age group (median grouping) and the SBP category, and between sex and the SBP category in the Cox regression models. We tested the proportional hazards assumption graphically and verified it using the Schoenfeld residual method. The assumption for proportionality was not violated.

We additionally performed sensitivity analyses after excluding participants with a DBP ≥ 80 mmHg (*n* = 11,897) or after excluding individuals who met the predefined categorical cut points to be free of CVD risk factors, but who had risk factor values in the upper normal range, i.e., (1) LDL-C level ≥ 130 mg/dL, or women with an HDL-C level < 50 mg/dL (*n* = 3164), and (2) blood glucose concentrations ≥ 100 mg/dL (*n* = 4122).

To examine whether changes in SBP and changes in the CVD risk factor parameters during the follow-up had an effect on the results, we eventually performed a time-dependent Cox regression analysis using the measurements of SBP and other CVD risk factors during the follow-up at visit #2 (2008–2009), visit #3 (2010–2011), visit #4 (2012–2013), visit #5 (2014–2015), and visit #6 (2016–2017). The participants were divided into five groups based on their SBP levels at each follow-up, namely 90–99 mmHg, 100–109, 110–119 mmHg, 120–129 mmHg, and ≥130 mmHg. In the time-dependent Cox regression, the follow-up time for each individual was divided into different short-time windows (each follow-up interval), a specific HR was calculated for each time window, and the weighted average of these window-specific HRs was determined. The traditional and time-dependent Cox regression analyses were performed again with a SBP level of 90–109 mmHg as the reference. We also calculated the rate of progression to systolic hypertension and the prevalence of CVD in different SBP groups at baseline. The SAS software (version 9.2, SAS Institute, Cary, NC, USA) was used for the statistical analyses. All statistical tests were two-sided, and a *P* value < 0.05 was considered statistically significant.

## Results

The study included 25,529 participants (15,297 (59.9%) men) with a mean age of 47.3 ± 12.3 (range: 18–95) years. The mean baseline SBP and mean baseline DBP were 114 ± 9.17 mmHg and 75.8 ± 7.50 mmHg, respectively. During the study period, the mean SBP and DBP were 119 ± 15.8 mmHg and 79.0 ± 9.47 mmHg, respectively, at 2 years after baseline, 121 ± 15.2 mmHg and 79.9 ± 9.42 mmHg, respectively, at 4 years after baseline, 122 ± 15.7 mmHg and 79.8 ± 9.32 mmHg, respectively, at 6 years after baseline, 128 ± 16.8 mmHg and 78.7 ± 10.0 mmHg, respectively, at 8 years after baseline, and 130 ± 16.9 mmHg and 77.8 ± 10.3 mmHg, respectively, at 10 years after baseline.

Analyzing the baseline examination data in univariate analysis, an increasing baseline SBP was associated with older age (*P* < 0.001), higher proportion of men (*P* < 0.001), lower level of education (*P* < 0.001), higher prevalence of ever smoking (*P* < 0.001), higher body mass index (*P* < 0.001), higher serum concentrations of total cholesterol (*P* < 0.001), low-density lipoproteins-C (*P* < 0.001), glucose (*P* < 0.001), C-reactive protein (*P* < 0.001) and uric acid (*P* < 0.001), higher prevalence of prediabetes, and lower eGFR (Table 1).

**Table 1:** Baseline characteristics of the study population.

	SBP, mmHg				
Parameter	90–99 (*n* = 1548)	100–109 (*n* = 4599)	110–119 (*n* = 8136)	120–129 (*n* = 11,246)	*P* for trend
Age, years	42.4 ± 12.0	44.4 ± 12.2	46.7 ± 12.1	49.5 ± 12.1	<0.001
Men, n(%)	532 (34.4)	2118 (46.1)	4789 (58.9)	7858 (69.9)	<0.001
BMI, kg/m^2^	22.7 ± 3.16	23.3 ± 3.22	24.0 ± 3.25	24.6 ± 3.27	<0.001
SBP, mmHg	93.2 ± 3.77	103 ± 3.72	112 ± 3.35	122 ± 2.83	<0.001
DBP, mmHg	63.1 ± 4.82	69.7 ± 5.58	75.5 ± 5.70	80.2 ± 5.64	<0.001
TC, mg/dL	182 ± 38.2	183 ± 35.9	186 ± 37.8	189 ± 40.1	<0.001
LDL, mg/dL	80.0 ± 28.2	82.0 ± 28.3	85.1 ± 29.8	84.9 ± 30.6	<0.001
HDL, mg/dL	60.9 ± 12.3	60.7 ± 12.7	60.8 ± 13.5	61.4 ± 14.0	0.003
Fbg, mg/dL	88.0 ± 11.0	88.5 ± 11.1	89.9 ± 11.6	91.1 ± 11.9	<0.001
CRP, mg/L	0.54 (0.20,1.70)	0.60 (0.21,1.80)	0.63 (0.23.1.74)	0.70 (0.25,1.94)	0.001
SUA, mg/dL	4.22 ± 1.18	4.42 ± 1.27	4.51 ± 1.29	4.61 ± 1.32	<0.001
eGFR, mL/min/1.73m^2^	88.1 ± 20.6	86.6 ± 22.1	85.8 ± 26.9	83.3 ± 25.1	<0.001
High school or above, *n* (%)	683 (45.4)	1685 (37.6)	2240 (28.4)	2060 (19.1)	<0.001
Ever smoking, *n* (%)	75 (4.8)	285 (6.2)	579 (7.1)	771 (6.9)	0.004
Exerciser, *n* (%)	1398 (93.1)	4163 (93.1)	7377 (93.7)	10145 (94.4)	0.013
Current drinker, *n* (%)	326 (21.5)	997 (22.1)	1699 (21.4)	2119 (19.6)	0.001
Prediabetes, *n* (%)	208 (13.4)	669 (14.5)	1524 (18.7)	2376 (21.1)	<0.001
Family history of CVD, *n* (%)	87 (5.6)	295 (6.4)	438 (5.4)	484 (4.3)	<0.001

SI conversion factors: To convert TC, HDL, and LDL to millimoles per liter, multiply by 0.0259; Fbg to millimoles per liter, multiply by 0.0555; SUA to micromoles per liter, divide by 0.0168.

*BMI* body mass index, *SBP* systolic blood pressure, *DBP* diastolic blood pressure, *TG* triglyceride, *TC* total cholesterol, *LDL* low-density lipoprotein, *HDL* high-density lipoprotein, *Fbg* fasting blood glucose, *CRP* C-reactive protein, *SUA* serum uric acid, *eGFR* estimated glomerular filtration rate, *CVD* cardiovascular disease.

During a mean follow-up of 10.6 ± 1.49 years (range: 0.04–11.5 years), 847 CVD events occurred. The rate of incident CVD events per 1000 person-years increased with increasing baseline SBP levels, with the baseline SBP levels of 90–99 mmHg, 100–109 mmHg, 110–119 mmHg, 120–129 mmHg having incidence of 1.45, 2.15, 3.06, and 3.80, respectively (Table 1).

**Fig. 1:**
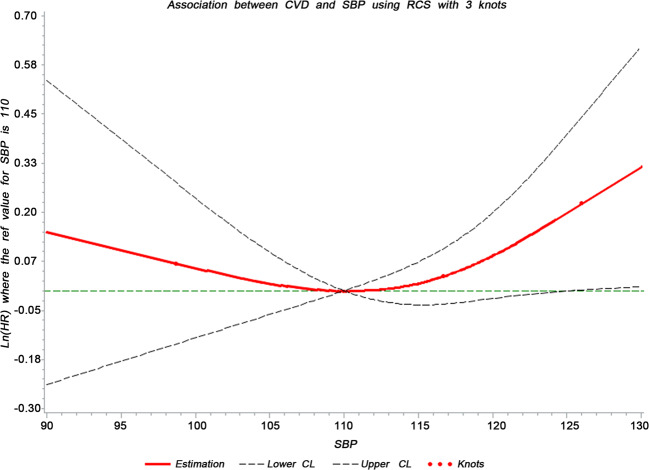
Adjusted Cubic Spline for the Hazard of Incident Cardiovascular Disease (CVD) by Systolic Blood Pressure (SBP. Adjusted for age, sex (male/female), alcohol consumption status (never and past, current, ≥1 time/day), ever smoking (yes/no), education level (elementary school, high school or above), exercise (none, occasionally or frequently, ≥1 times/week), prediabetes (yes/no), family history of cardiovascular diseases (yes/no), diastolic blood pressure, body mass index, serum concentrations of total cholesterol, high-density lipoprotein cholesterol, glucose, uric acid and C-reactive protein, and estimated glomerular filtration rate.

After adjusting for age, sex, alcohol consumption status, status of ever smoking, level of education, physical exercise, prediabetes, family history of CVD, DBP, body mass index, serum concentrations of total cholesterol, high-density lipoproteins-C, glucose, uric acid and C-reactive protein, and eGFR, the cubic spline interpolation revealed that the relationship between different levels of baseline SBPs values within the normal range and the CVD risk showed a U-shaped curve, with the lowest point corresponding to a baseline SBP of 110 mmHg (Fig. 1). However, statistically significant results were demonstrated only for SBP values >125 mmHg, while there was no statistical significance for SBP values <125 mmHg (Fig. 1). The categorical Cox regression further suggested that the CVD risk was not statistically significant for study participants with a baseline SBP level of 100–109 mmHg, 110–119 mmHg, and 120–129 mmHg compared with those with a baseline SBP level of 90–99 mmHg, after adjusting for the parameters listed above (Table 2; Supplementary Tables 1, 2). The Cox regression model also showed that there was no significant interaction between age and SBP category and between sex and SBP category, so we did not perform further age or sex subgroup analysis.

**Table 2:** Hazard ratios of cardiovascular events by systolic blood pressure categories within the normal range.

Parameter	SBP, mmHg			
	90–99	100–109	110–119	120–129
Case/n	24/1548	106/4599	265/8136	452/11246
Incidence/1000 person-years	1.45	2.15	3.06	3.80
Model 1	Ref.	1.48 (0.95–2.30)	2.10 (1.38–3.18)^a^	2.60 (1.72–3.92)^a^
Model 2	Ref.	1.20 (0.77–1.88)	1.43 (0.94–2.18)	1.45 (0.96–2.19)
Model 3	Ref.	1.12 (0.69–1.80)	1.21 (0.75–1.93)	1.09 (0.67–1.77)
Model 4	Ref.	1.16 (0.71–1.89)	1.25 (0.78–2.02)	1.13 (0.69–1.86)

Model 1: unadjusted. Model 2: adjusted for age and sex (male/female). Model 3: adjusted for age, sex (male/female), alcohol consumption status (never and past, current, ≥1 time/day), ever smoking (yes/no), education level (elementary school, high school or above), exercise (none, occasionally or frequently, ≥1 times/week), prediabetes (yes/no), family history of cardiovascular diseases (yes/no), diastolic blood pressure, body mass index, serum concentrations of total cholesterol, high-density lipoprotein cholesterol, glucose, uric acid and C-reactive protein, and estimated glomerular filtration rate. Model 4 was a competing risk model of death which adjusted for all the confounders in model 3. Compared with reference group*.*

^a^*P*  <  0.01.

The results remained overall unchanged if we additionally excluded participants with a DBP level ≥ 80 mmHg or participants who had risk factor values outside the normal range (i.e., serum concentrations of low-density lipoproteins-C ≥ 130 mg/dL, serum glucose concentration ≥ 100 mg/dL, or women with a serum concentration of high-density lipoprotein-C level <50 mg/dL) (Tables 3, 4). If participants with a DBP level ≥ 80 mmHg and participants with risk factor values outside the normal range were excluded, the associations between a baseline SBP of 100–109 mmHg, 110–119 mmHg, and 120–129 mmHg and CVD incidence were still not significant (Table 5).

**Table 3:** Hazard ratios of cardiovascular events stratified by baseline systolic blood pressure categories in the normal range, after further excluding participants who had a DBP ≥ 80 mmHg.

Characteristic	SBP, mmHg			
	90–99	100–109	110–119	120–129
Case/N	24/1545	96/4189	161/5158	108/2740
Incidence/1000 person-years	1.46	2.14	2.94	3.76
Model 1	Ref.	1.47 (0.94–2.30)	2.01 (1.31–3.09)^a^	2.57 (1.65–4.00)^a^
Model 2	Ref.	1.23 (0.78–1.92)	1.35 (0.88–2.07)	1.19 (0.76–1.87)
Model 3	Ref.	1.22 (0.75–1.99)	1.28 (0.78–2.11)	1.06 (0.62–1.79)
Model 4	Ref.	1.25 (0.76–2.08)	1.32 (0.79–2.22)	1.10 (0.64–1.89)

Model 1: unadjusted. Model 2: adjusted for age and sex (male/female). Model 3: adjusted for age, sex (male/female), alcohol consumption status (never and past, current, ≥1 time/day), ever smoking (yes/no), education level (elementary school, high school or above), exercise (none, occasionally or frequently, ≥1 times/week), prediabetes (yes/no), family history of cardiovascular diseases (yes/no), diastolic blood pressure, body mass index, serum concentrations of total cholesterol, high-density lipoprotein cholesterol, glucose, uric acid and C-reactive protein, and estimated glomerular filtration rate. Model 4 was a competing risk model of death which adjusted for all the confounders in model 3. Compared with reference group.

^a^*P*  <  0.01.

**Table 4:** Hazard ratios of cardiovascular events stratified by baseline normal systolic blood pressure categories in the normal range, after further excluding participants who had risk factor values above normal (i.e., serum concentrations of low-density lipoproteins-C ≥ 130 mg/dL, serum glucose concentration ≥ 100 mg/dL, or women with a serum concentration of high-density lipoprotein-C level < 50 mg/Dl).

Characteristic	SBP, mmHg			
	90–99	100–109	110–119	120–129
Case/N	21/1158	76/3407	195/5790	305/7888
Incidence/1000 person-years	1.70	2.08	3.17	3.65
Model 1	Ref	1.22 (0.75–1.98)	1.86 (1.18–2.91)^a^	2.14 (1.37–3.32)^a^
Model 2	Ref	1.00 (0.62–1.62)	1.29 (0.82–2.03)	1.20 (0.77–1.88)
Model 3	Ref	0.90 (0.53–1.52)	1.10 (0.65–1.85)	0.89 (0.52–1.53)
Model 4	Ref	0.94 (0.55–1.60)	1.14 (0.67–1.93)	0.92 (0.53–1.60)

Model 1: unadjusted. Model 2: adjusted for age and sex (male/female). Model 3: adjusted for age, sex (male/female), alcohol consumption status (never and past, current, ≥1 time/day), ever smoking (yes/no), education level (elementary school, high school or above), exercise (none, occasionally or frequently, ≥1 times/week), family history of cardiovascular diseases (yes/no), diastolic blood pressure, body mass index, serum concentrations of total cholesterol, high-density lipoprotein cholesterol, glucose, uric acid and C-reactive protein, and estimated glomerular filtration rate. Model 4 was a competing risk model of death which adjusted for all the confounders in model 3. Compared with reference group.

^a^*P*  <  0.01.

**Table 5:** Hazard ratios of cardiovascular events stratified by baseline systolic blood pressure categories in the normal range, after excluding participants who had a diastolic blood pressure ≥ 80 mmHg and had risk factor values above normal (i.e., serum concentrations of low-density lipoproteins-C ≥ 130 mg/dL, serum glucose concentration ≥100 mg/dL, or women with a serum concentration of high-density lipoprotein-C level < 50 mg/Dl).

Characteristic	SBP, mmHg			
	90–99	100–109	110–119	120–129
Case/N	21/1156	68/3121	116/3667	73/1941
Incidence/1000 person-years	1.70	2.03	2.98	3.57
Model 1	Ref.	1.19 (0.73–1.95)	1.74 (1.10–2.77)^b^	2.09 (1.29–3.39)^a^
Model 2	Ref.	1.00 (0.61–1.63)	1.19 (0.74–1.89)	0.95 (0.58–1.55)
Model 3	Ref.	0.98 (0.57–1.70)	1.19 (0.68–2.07)	0.88 (0.48–1.59)
Model 4	Ref.	1.02 (0.58–1.78)	1.22 (0.69–2.16)	0.91 (0.50–1.68)

Model 1: unadjusted. Model 2: adjusted for age and sex (male/female). Model 3: adjusted for age, sex (male/female), alcohol consumption status (never and past, current, ≥1 time/day), ever smoking (yes/no), education level (elementary school, high school or above), exercise (none, occasionally or frequently, ≥1 times/week), family history of cardiovascular diseases (yes/no), diastolic blood pressure, body mass index, serum concentrations of total cholesterol, high-density lipoprotein cholesterol, glucose, uric acid and C-reactive protein, and estimated glomerular filtration rate. Model 4 was a competing risk model of death which adjusted for all the confounders in model 3. Compared with reference group*.*

^a^*P* <  0.01.

^b^*P*  <  0.05.

Finally, since participants with a normal SBP at baseline may develop hypertension during the follow-up, we entered the BP category as a time-dependent explanatory variable in a time-dependent Cox regression analysis, in which at each point in time, the last available BP measurement was considered (Supplementary Tables 3, 4). In a similar manner, the parameter of intake of antihypertensive drugs and other covariates that changed over time were entered into the model as time-dependent covariates. It revealed that the normal baseline SBP was still not associated with incident CVD, neither in the group of a baseline SBP of 120–129 mmHg or in any other group. In addition, with a SBP level of 90–109 mmHg as the reference, both the traditional and time-dependent Cox regression model suggested that the baseline SBP was not significantly associated with the incidence of CVDs.

## Discussion

The current study found that the CVD incidence increased with higher baseline SBP level in the normotensive population without hypertension and without other cardiovascular risk factors at baseline. After adjusting for risk factors, the relationship between higher baseline SBP (within the normal range) and higher CVD risk showed a U-shaped curve. However, statistically significant results were demonstrated only for SBP values > 125 mmHg, while there was no statistical significance for SBP values <125 mmHg. The categorical Cox regression further suggested that the CVD risk was not statistically significant for study participants with a baseline SBP level of 100–109 mmHg, 110–119 mmHg, and 120–129 mmHg compared with those with a baseline SBP level of 90–99 mmHg, after adjusting for the parameters listed above.

In contrast to our study, the MESA reported on a dose-effect relationship between baseline SBP level and CVD risk in a population without hypertension and without other cardiovascular risk factors [8]. The reasons for the discrepancy between both studies may be differences in the study population, sample size (*n* = 1457 versus *n* = 25,529), age (mean age 58 years versus 47 years), and rate of CVD events (6.5% (MESA) versus 2.7% in our study) and the inclusion of BP measurements and other CVD risk factors, examined during the follow-up period, into the statistical analysis in our study. In other study populations, a baseline SBP between 120 and 129 mmHg as compared with normal SBP (SBP < 120 mmHg) increased the CVD risk, however, traditional cardiovascular risk factors such as diabetes and dyslipidemia were not fully excluded [21–24].

Since an association between isolated diastolic hypertension and incident CVD has not unequivocally been confirmed, participants with a DBP ≥ 80 mmHg were not excluded in the initial analysis in our study [25, 26]. Interestingly, the results of our analysis did not markedly change after excluding study participants with a DBP ≥ 80 mmHg in a sensitivity analysis. In a similar manner, when we excluded participants with diabetes or dyslipidemia, the results of the analysis did not change. When we excluded both, individuals with isolated diastolic hypertension and participants with other cardiovascular risk factors, the adjusted HR for CVD was neither statistically significant (Table 3). One of the reasons for that finding may have been the reduction in the number of CVD events limiting the statistical power of the analysis.

We also observed that the baseline SBP was not correlated with the CVD incidence when the BP category as a time-dependent explanatory variable was included in a time-dependent Cox regression analysis. However, the proportion of participants who eventually progressed to hypertension gradually increased with the baseline SBP categories. Correspondingly, the levels of traditional cardiovascular risk factors such as body mass index, serum concentrations of total cholesterol, low-density lipoprotein-C, glucose, C-reactive protein and uric acid, increased with higher baseline SBP within the normal range. It suggests that individuals with elevated SBP should be monitored closely for the eventual development of CVD risk factors which may then cause an increased incidence of CVD. It holds true in particular since studies have shown that the cumulative effects of these CVD risk factors increase the CVD risk [27–29].

The clinical implications of results of our study are first, that a SBP between 90 and 129 mmHg is not associated with an increased CVD risk in a healthy population. Second, even in the healthy population, some traditional cardiovascular risk factors still presented a trend of aggregation with the increase of baseline SBP level. Therefore, we advise prevention and control of cardiovascular risk factors through dietary and lifestyle improvements to reduce the cardiovascular risk in the healthy population.

When the results of our study are discussed, its limitations should be taken into account. First, our study was not an intervention trial and has just shown associations. Second, although the participants with a SBP of 120–129 mmHg did not have any medical problems, they showed a trend toward CVD risk factors, what might have influenced the development of CVDs (Table 1). Third, we did not include all cardiovascular risk factors, such as high-salt diet and air pollution, into our analysis. Fourth, the CVD rate in our study population was lower than in the MESA study population, perhaps due to that our study participants were mainly employed, had a younger age and that the follow-up was shorter. The CVD rate might therefore have been underestimated in our study. Fifth, in agreement with the MESA study, the proportion of women decreased and the mean age increased with higher SBP category. Since the CVD incidence depends on age and sex, the uneven distribution of both parameters might have led to a bias. Further statistical analysis however did not show a significant interaction between age or sex and the SBP categories. Sixth, the individuals with a baseline SBP of 120–129 mmHg were older than those with a baseline SBP of 90–110 mmHg (49.5 ± 12.1 years versus 42.4 ± 12.0 years; *P* < 0.001), so that one may argue that this age difference at the baseline examination and not the BP might have been responsible for the noted outcomes. In the multivariable analysis, however, age was included into the list of independent variables, and the results remained to be statistically significant. Strengths of our study include the study sample size, the relatively high number of parameters and risk factors assessed, the repeated measures of the CVD risk factors, the standardized data collection protocols, and a mostly complete follow-up for the detection of CVD events, since the Municipal Social Insurance collected all medical records of the entire population of the Kailuan community.

In conclusion, in this population without hypertension and without other traditional cardiovascular risk factors at baseline, although SBP levels were not found to be associated with CVD risk, individuals with an elevated baseline SBP level of 120–129 mmHg had indirectly a higher CVD risk through a higher chance of developing cardiovascular risk factors such as hypertension. Individual with a high-normal SBP of 120–129 mmHg may be closely followed for an early detection of a BP rise as main risk factor for incident CVDs.

### Summary

#### What is known about the topic

- High BP as an important risk factor for CVDs is a leading cause of disability and death. It has remained unclear whether the association between the SBP level and the CVD risk is valid also among healthy normotensive adults without traditional cardiovascular risk factors.
- The MESA reported that normotensive participants without conventional cardiovascular risk factors showed during an average follow-up of 14.5 years an increase in the CVD risk by 53% for every 10 mmHg increase in the baseline SBP. It suggested a dose-effect rather than a J-shaped or U-shaped relationship between the SBP level and CVD risk in a healthy population.
- Since risk factors differ between regions and societies, we performed this study to investigate the association between the baseline SBP level within the normal range and the CVD risk in a normotensive population without conventional cardiovascular risk factors in China.

#### What this study adds

- In this cohort study including 25,529 participants without CVD at baseline (follow-up: 10.6 ± 1.5 years), the CVD incidence per 1000 person-years increased in univariate analysis with higher baseline SBP levels within the normotensive range.
- After adjusting for CVD risk factors, the CVD risk was not statistically significant for normotensive study participants with a baseline SBP level of 100 mmHg or higher as compared with those with a baseline SBP level of 90–99 mmHg.
- As a corollary, if CVD risk factors including BP categories which developed during follow-up were included in a time-dependent Cox regression analysis, the normal baseline SBP was neither associated with incident CVDs.

## Supplementary information

Supplemental Tables
