# Supplementary material for: Level of systolic blood pressure within the normal range and risk of cardiovascular events in the absence of risk factors in Chinese
Source: J Hum Hypertens. 2021 Sep 3;36(10):933–9. doi: 10.1038/s41371-021-00598-1 (PMC9553643; doi:10.1038/s41371-021-00598-1)
Supplement: Supplementary file 1 — Supplemental Tables [file 41371_2021_598_MOESM1_ESM.docx]

Supplementary Table 1 Hazard ratios of cardiovascular events by systolic blood pressure categories (with group of 90-109 mmHg as reference) within the normal range

| Parameter | SBP, mmHg | | |
| --- | --- | --- | --- |
|  | 90-109 | 110-119 | 120-129 |
| Case / n | 24/1548 | 265/8136 | 452/11246 |
| Incidence/1000 person-years | 1.45 | 3.06 | 3.80 |
| Model 1 | Ref | 1.55 (1.25-1.91)^a^ | 1.91 (1.57-2.33)^a^ |
| Model 2 | Ref | 1.24 (1.00-1.52)^b^ | 1.25 (1.03-1.52)^b^ |
| Model 3 | Ref | 1.10 (0.87-1.39) | 0.99 (0.77-1.27) |
| Model 4 | Ref | 1.10 (0.87-1.39) | 0.99 (0.78-1.27) |

Abbreviations: Model 1: unadjusted. Model 2: adjusted for age and sex (male/female). Model 3: adjusted for age, sex (male/female), alcohol consumption status (never and past, current, ≥1 time/day), ever smoking (yes/no), education level (elementary school, high school or above), exercise (none, occasionally or frequently, ≥1 times/week), prediabetes (yes/no), family history of cardiovascular diseases (yes/no), diastolic blood pressure, body mass index, serum concentrations of total cholesterol, high-density lipoprotein cholesterol, glucose, uric acid and C-reactive protein, and estimated glomerular filtration rate. Model 4 was a competing risk model of death which adjusted for all the confounders in model 3. Compared with reference group, ^a^*P*<0.01.

Supplementary Table 2 Associations of the incidence of cardiovascular events with baseline systolic blood pressure categories (with group of 90-109mmHg as reference) within the normal range among Chinese adults with time-dependent variables

| Characteristic | SBP, mmHg | | | |
| --- | --- | --- | --- | --- |
|  | 90-109 | 110-119 | 120-129 | ≥130 |
| Model 1 | Ref | 1.84 (1.38-2.45)^a^ | 2.19 (1.67-2.88)^a^ | 4.24 (3.25-5.52)^a^ |
| Model 2 | Ref | 1.21 (0.88-1.67) | 1.16 (0.85-1.58) | 1.78 (1.32-2.40)^a^ |
| Model 3 | Ref | 1.03 (0.73-1.47) | 0.97 (0.68-1.37) | 1.25 (0.86-1.83) |

Abbreviations: Model 1: unadjusted. Model 2: adjusted for age and sex (male/female). Model 3: adjusted for age, sex (male/female), alcohol consumption status (never and past, current, ≥1 time/day), ever smoking (yes/no), education level (elementary school, high school or above), exercise (none, occasionally or frequently, ≥1 times/week), prediabetes (yes/no), family history of cardiovascular diseases (yes/no), diastolic blood pressure, body mass index, serum concentrations of total cholesterol, high-density lipoprotein cholesterol, glucose, uric acid and C-reactive protein, and estimated glomerular filtration rate. Compared with reference group, ^a^*P*<0.01.

Supplementary Table 3 Changes in blood pressure levels and prevalence of cardiovascular events during the follow-up period

| Baseline SBP, mmHg | Follow-up | | | |
| --- | --- | --- | --- | --- |
|  | Systolic hypertension | | Normal SBP | |
|  | N(%) | CVD, n(%) | N(%) | CVD, n(%) |
| 90-99 | 469(30.3) | 10(2.13) | 1079(69.7) | 14(1.30) |
| 100-109 | 2005(43.6) | 64(3.19) | 2594(56.4) | 42(1.62) |
| 110-119 | 4638(57.0) | 185(3.99) | 3498(43.0) | 80(2.29) |
| 120-129 | 8061(71.7) | 354(4.39) | 3185(28.3) | 98(3.08) |

Supplementary Table 4 Associations of the incidence of cardiovascular events with baseline systolic blood pressure categories within the normal range among Chinese adults with time-dependent variables

| Characteristic | SBP, mmHg | | | | |
| --- | --- | --- | --- | --- | --- |
|  | 90-99 | 100-109 | 110-119 | 120-129 | ≥130 |
| Model 1 | Ref | 1.09 (0.61-1.93) | 1.96 (1.16-3.33)^b^ | 2.33 (1.39-3.93)^a^ | 4.51 (2.69-7.56)^a^ |
| Model 2 | Ref | 0.75 (0.41-1.37) | 0.97 (0.55-1.68) | 0.93 (0.54-1.60) | 1.42 (0.83-2.45) |
| Model 3 | Ref | 0.73 (0.37-1.43) | 0.80 (0.42-1.50) | 0.75 (0.39-1.41) | 0.96 (0.50-1.86) |

Abbreviations: Model 1: unadjusted. Model 2: adjusted for age and sex (male/female). Model 3: adjusted for age, sex (male/female), alcohol consumption status (never and past, current, ≥1 time/day), ever smoking (yes/no), education level (elementary school, high school or above), exercise (none, occasionally or frequently, ≥1 times/week), prediabetes (yes/no), family history of cardiovascular diseases (yes/no), use of anti-hypertension medicines (yes/no), diastolic blood pressure, body mass index, serum concentrations of total cholesterol, high-density lipoprotein cholesterol, glucose, uric acid and C-reactive protein, and estimated glomerular filtration rate. Compared with reference group, ^a^*P*<0.01.
